# Commentary: Managing Thoracic Aortic Aneurysm in Patients with Bicuspid Aortic Valve Based on Aortic Root-Involvement

**DOI:** 10.3389/fphys.2017.00838

**Published:** 2017-10-30

**Authors:** Marco Russo, Paolo Nardi, Guglielmo Saitto, Fabio Bertoldo, Giovanni Ruvolo

**Affiliations:** Department of Cardiac Surgery, Tor Vergata University of Rome, Rome, Italy

**Keywords:** bicuspid valve, ascending aorta, aortic root, valve sparing, genetic syndromes

We read with great interest the paper from Norton and Yang (2017) in which they optimally described their philosophy in the treatment of patients affected by concomitant bicuspid aortic valve and thoracic aorta dilation. They described an interesting tailored approach based on the involvement of aortic root: an aggressive surgical resection for diameter larger than 50 mm is proposed in case of root dilation associated with aortic insufficiency (Cluster A; malignant form) whereas a less precocious aortic resection (diameter > 55 mm) for “cluster B” patients, defined as no root dilation but with ascending aorta enlargement (benign form).

Patients affected by bicuspid aortic valve aortophaty represent a heterogeneous population in which both genetics and hemodynamic factors play a special role in the pathogenesis of proximal aorta dilation (Veldtman et al., 2006; Michelena et al., 2008; Della Corte et al., 2014).

In this brief commentary we would like to focus on a particular subgroup of patients, that we would like to define as “modified Cluster B”: in this population the dilation of ascending aorta represents the indication for operation in presence of a normally functioning bicuspid aortic valve and not dilated root. These patients represent a very rare cohort in which the cardiac surgeon should manage the possibility to preserve a not-malfunctioning but congenitally altered valve and with not enlarged bicuspid Sinues of Valsalva.

In our recently published data (Russo et al., 2017) on 47 patients (mean age 57 ± 11 years, 31 males) treated in a 20-years period, we reported a very low risk of reoperation on spared aortic root with an actuarial freedom reoperation of 100 and 94.4 ± 5.6% at 5 and 10 years. No significantly enlargement of Sinuses was recorded in echo assessment and no new acute aortic syndromes occurred. Moreover, the requirement for a reoperation during the follow-up period was determined by leaflet pathology in presence of still normal root. We assessed that, even in the setting of a bicuspid aortic root, the treatment of a cluster B patient with normally functioning valve and not enlarged Sinues is a safe and valuable option (Russo et al., 2017). Moreover we observed that this particular aortic dilation phenotype is associated with type 0 BAV with antero-posterior orientation. Maybe this association described a particular subcategory with a genetic substrate not yet completely understood.

What we would like to know is the Author's experience when faced with a similar situation of “modified Cluster B” patients.

What could we learn more in the field of bicuspid aortic valve syndrome?

## Author contributions

MR is the main investigator. PN was responsible of data analysis. GS participated to data collection of follow up. FB was responsible of data collection. GR was coordinator.

### Conflict of interest statement

The authors declare that the research was conducted in the absence of any commercial or financial relationships that could be construed as a potential conflict of interest.
